# Traitement arthroscopique d'une fracture articulaire de la glène: nouvelle astuce

**DOI:** 10.11604/pamj.2015.20.268.6433

**Published:** 2015-03-19

**Authors:** Samir Hamoudi, Ihab Alassaf, Hassan Boussakri, Philbert Ntrataze, Jean François Dumez

**Affiliations:** 1 Service de Chirurgie Orthopédique et Traumatologique(AP4), CH Moulins, France

**Keywords:** Glène, fracture, arthroscopie, fixation, glenoid, fracture, arthroscopy, fixation

## Abstract

Le traitement des fractures de la glène scapulaire constitue un sujet de débat dans la littérature. Les auteurs décrivent une observation d'un patient âgé de 22 ans qui présente une fracture articulaire de la glène classée stade III de Ideberg traitée chirurgicalement sous arthroscopie. Nous exposons une nouvelle technique chirurgicale utilisant un matériel simple et nous la recommandons pour ce type de fracture qui constitue une alternative efficace. Globalement, nos résultats cliniques et anatomiques immédiats et à moyen terme, au dernier recul, sont excellents.

## Introduction

Les fractures de la glène scapulaire constituent un sujet de débat dans la littérature [1]. Elles sont, généralement, secondaires à un traumatisme de haute énergie. Plusieurs classifications sont décrites pour codifier la prise en charge thérapeutique de cette entité pathologique rare. La plus utilisée est la classification d'IDEBERG [2–4]. Son traitement chirurgical reste le sujet d'actualité avec le développement de l'exploration arthroscopique de l’épaule. Le type Ideberg III est rare, sa prise en charge thérapeutique reste difficile et la majorité des auteurs préconisent une réduction à ciel ouvert avec fixation interne ORIF [5]. L'approche artroscopique était décrite, mais surtout pour des fractures antérieures de la glène utilisant des techniques de suture pour le contrôle et la fixation du fragment, rarement des techniques de fixation par vissage percutané [6–8]. Nous rapportons un cas de fracture articulaire de la glène type III d'Ideberg chez un jeune patient de 22 ans suite à une chute de moto. Le but de ce travail est de présenter notre expérience à travers cette observation ainsi qu'une nouvelle astuce simple, utilisant un matériel disponible dans toutes les salles opératoires d'orthopédie. Globalement nos résultats anatomo-cliniques sont excellents.

## Patient et observation

**Figure 1:**
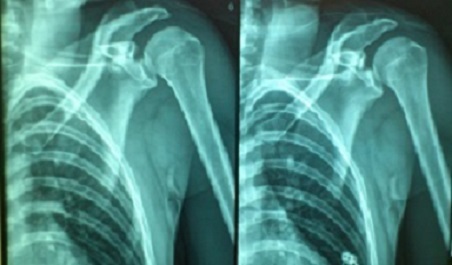
Radiographie standard de l’épaule gauche montre une fracture articulaire de la glène scapulaire

**Figure 2:**
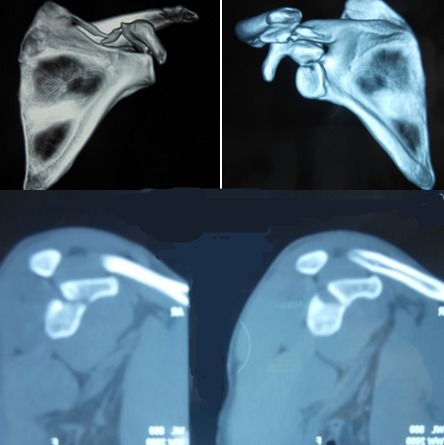
Coupes scannographiques avec reconstruction tridimensionnelles (3D) montrant une fracture stade III de Ideberg

**Figure 3:**
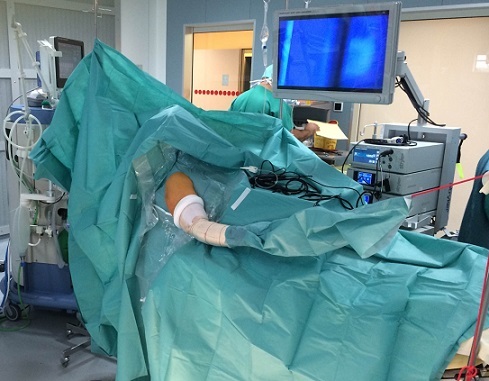
Installation classique d'arthroscopie

Monsieur M.R âgé de 22 ans droitier, travailleur manuel de son état, victime le jour de son admission d'un accident de moto à haute cinétique par glissade. Le patient portait une tenue de protection ainsi que son casque. Il a été pris en charge rapidement par l’équipe de SMUR et évacué vers notre établissement. Le bilan clinique, après avoir éliminé une urgence vitale, mettait en évidence un traumatisme de l’épaule gauche avec à l'examen une impotence fonctionnelle totale et douloureuse, sans déficit vasculaire ni nerveux du membre supérieur; un traumatisme fermé du bassin et un traumatisme fermé de la main droite avec. Le bilan clinique était complété par un bilan radiologique avec scanner et reconstruction 2D et 3D qui objectivait une fracture de la glène à trait transversal détachant un fragment supérieur dont la taille est d'environ 1/3 de la surface articulaire type III de IDEBERG (Figure 1, Figure 2); une fracture des deux branches Ilio-pubiennes droite et gauche; une fracture de Bennett droite, La prise en charge chirurgicale par arthroscopie a été planifiée après analyse des résultats cliniques et radiologiques par une équipe de chirurgiens entrainées et spécialisées à la chirurgie de l’épaule. **Technique opératoire:** malade sous anesthésie générale, installation opératoire en beach-chair, position semi-assise, avec traction non collée de 1 Kg du membre supérieur gauche. Colonne d'arthroscopie et source radiologique à proximité; l'amplificateur de brillance était installé en arceau au-dessus de l’épaule (Figure 3).

**Figure 4:**
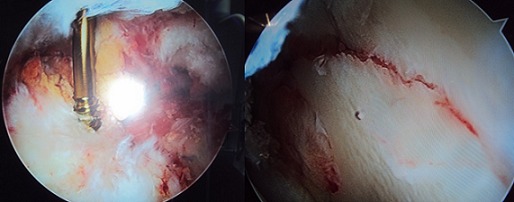
Aspects arthroscopiques peropératoires. a): mise en place de la vis canulée après réduction. b): Réduction anatomique de la fracture après ostéosynthèse interne

**Figure 5:**
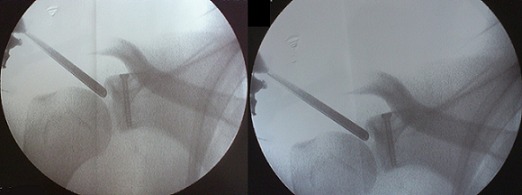
Contrôle radiologique peropératoire (par amplificateur de brillance) de la réduction après fixation de la fracture

**Figure 6:**
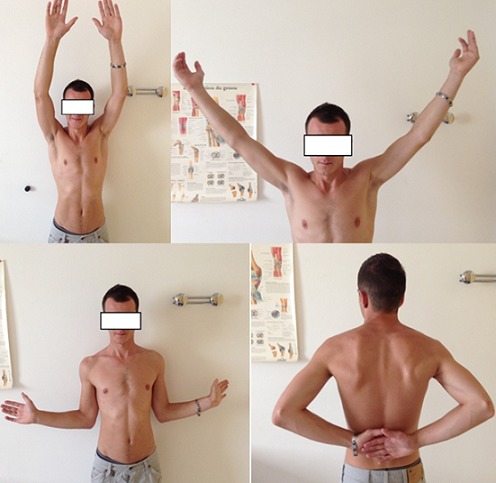
Amplitudes articulaires normales au dernier recul

**Figure 7:**
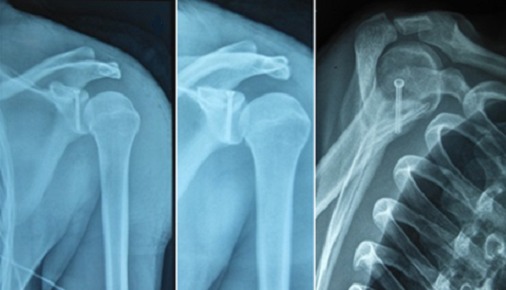
Résultats radiologiques satisfaisant après un recul de 6 mois

Après aseptisation du champ opératoire par badigeonnage et mise en place conventionnelle des champs, nous avons utilisé une voie d'abord postérieure habituelle qui nous permet la visualisation de la cavité gléno-humérale avec la mise en évidence d'une fracture à trait transversal à l'union 1/3 supérieur 1/3 moyen de la glène. La manipulation du fragment proximal était facilitée par l'utilisation d'une première broche de kirschner supérieure passée en per cutanée et qui traverse légèrement ce dernier au niveau du foyer de fracture, ce qui fournit un point d'appui pour le crochet. Alors, on réussit une très bonne réduction avec une stabilisation première en utilisant la même broche, puis définitive avec une vis canulée diamètre 4,5 guidée par une 2^ème^ broche de Kirschner qui est mise dans l'axe de la glène, perpendiculaire au trait de fracture et parallèle à la surface articulaire. Un contrôle par l'amplificateur de brillance confirme le bon positionnement et la bonne longueur de la vis. Sur le contrôle optique on obtient une réduction quasi anatomique de la surface articulaire avec un effet de compression (Figure 4, Figure 5); nous avons terminé l'intervention par un brochage type Iselin du côté droit. Dans les suites immédiates, le patient était immobilisé dans une contention coude au corps qu'il a gardé 3 semaines. A l'issu de cette période nous avons entamé une rééducation fonctionnelle de l’épaule qui s'est déroulée en trois phases: d'abord antalgique suivie d'une mobilisation passive puis active avec renforcement musculaire. L’évolution fonctionnelle était très satisfaisante avec un score de DASH qui passe de 40 à 2 mois post opératoire avec une dizaine de séances de rééducation fonctionnelles, à 3 après une quarantaine de séances de rééducation fonctionnelles avec une mobilité et une force musculaire quasi complète (Figure 6). Sur le plan radiologique, on note une consolidation quasi complète de sa fracture (Figure 7).

## Discussion

La glèno-humérale est l'articulation du corps la plus mobile et présente des degrés de liberté assez importants permettant de porter la main dans toutes les zones de l'espace; d'autant plus que la proximité du plexus brachial et des vaisseaux axillaires impose un examen vasculo-nerveux précis et l'utilisation d'une technique chirurgicale fiable et rassurante. Les fractures scapulaires ont une incidence annuelle de 10/10^5^, la glène est touchée dans 30% des cas [9]. Ce sont des fractures secondaires à des traumatismes violents avec choc direct sur le moignon de l’épaule. Les manifestations cliniques sont immédiates avec impotence fonctionnelle totale et douloureuse du membre supérieur atteint. Un bilan clinique est indispensable à la recherche des complications vasculo-nerveuses, il sera complété par un bilan radiologique notamment la TDM avec les reconstructions 3D qui permettent de mieux classer la fracture et codifier une stratégie thérapeutique adéquate. Le but du traitement est d'obtenir une épaule mobile, stable et indolore avec le moins de complications. Etant donné que ce sont des fractures articulaires aucun défaut de réduction ne sera toléré et donc le traitement ne pourra être que chirurgical. Le choix doit se poser entre la chirurgie à ciel ouvert et l'arthroscopie, nous avons opté pour une approche arthroscopique. Après une planification radio-clinique et instrumentale; l'intervention s'est déroulée avec une installation d'arthroscopie standard en position semi-assise, un amplificateur de brillance placé en arceau, par voies d'abords habituelles. Notre astuce utilisant un matériel simple et disponible (broches de kirschner 1,5) nous a permis d'obtenir une réduction parfaite de la fracture qu'on contrôle par la vue (arthroscopie) et stabiliser par une vis cannelée mise en place en percutanée par voie supérieur sous contrôle d'amplificateur de brillance. Néanmoins cette technique présente des limites à savoir les fractures à plusieurs fragments, ou les fractures à petits fragments non synthésables par des vis ainsi que le type I et le type II de Ideberg ou le vissage percutané par voie antérieure risque d’être dangereux sur les paquets vasculo-nerveux [10].

Comparé à notre technique, les ORIF sont les plus utilisés, notamment la voie postérieure, avec des résultats fonctionnels qui sont satisfaisants, mais c'est une technique qui reste délabrante avec un risque de complication décrit qui reste rare à savoir l'infection, raideur de l’épaule et la défaillance du matériel. La rééducation fonctionnelle constitue un volet primordial de la prise en charge thérapeutique dans ce genre de traumatisme et doit être débutée rapidement après une période d'immobilisation plus au mois courte.

## Conclusion

Nous pensons que notre nouvelle technique va faciliter le vissage percutané des fractures de la glène type Ideberg III avec une approche arthroscopique, dont les résultats à moyen terme de notre cas sont excellents.
